# Migration of primordial germline cells is negatively regulated by surrounding somatic cells during early embryogenesis in Drosophila melanogaster

**DOI:** 10.18699/VJ20.644

**Published:** 2020-08

**Authors:** N.V. Dorogova, A.S. Khruscheva, Iu.A. Galimova, D.Yu. Oshchepkov, D.E. Maslov, E.D. Shvedkina, K.A. Akhmetova, S.A. Fedorova

**Affiliations:** Institute of Cytology and Genetics of Siberian Branch of the Russian Academy of Sciences, Novosibirsk, Russia; Institute of Cytology and Genetics of Siberian Branch of the Russian Academy of Sciences, Novosibirsk, Russia; Institute of Molecular and Cellular Biology of Siberian Branch of the Russian Academy of Sciences, Novosibirsk, Russia; Institute of Cytology and Genetics of Siberian Branch of the Russian Academy of Sciences, Novosibirsk, Russia; Novosibirsk State University, Novosibirsk, Russia; Novosibirsk State University, Novosibirsk, Russia; Institute of Cytology and Genetics of Siberian Branch of the Russian Academy of Sciences, Novosibirsk, Russia University of Alabama at Birmingham, Department of Biochemistry and Molecular Genetics, School of Medicine, Birmingham, Alabama, USA; Institute of Cytology and Genetics of Siberian Branch of the Russian Academy of Sciences, Novosibirsk, Russia

**Keywords:** Drosophila melanogaster, embryogenesis, germline cells, cell migration, embryonic gonad development, Drosophila melanogaster, эмбриогенез, клетки зародышевой линии, миграция клеток, формирование эмбриональных гонад

## Abstract

Cell migration is an important morphogenetic process necessary at different stages of individual development and body functioning. The initiation and maintenance of the cell movement state requires the activation of many factors involved in the regulation of transcription, signal transduction, adhesive interactions, modulation of membranes and the cytoskeleton. However, cell movement depends on the status of both migrating and surrounding cells, interacting with each other during movement. The surrounding cells or cell matrix not only form a substrate for movement, but can also participate in the spatio-temporal regulation of the migration. At present, there is no exact understanding of the genetic mechanisms of this regulation. To determine the role of the cell environment in the regulation of individual cell migration, we studied the migration of primordial germline cells (PGC) during early embryogenesis in Drosophila melanogaster. Normally, PGC are formed at the 3rd stage of embryogenesis at the posterior pole of the embryo. During gastrulation (stages 6–7), PGC as a consolidated cell group passively transfers into the midgut primordium. Further, PGC are individualized, acquire an amoeboid form, and actively move through the midgut epithelium and migrate to the 5–6 abdominal segment of the embryo, where they form paired embryonic gonads. We screened for genes expressed in the epithelium surrounding PGC during early embryogenesis and affecting their migration. We identified the myc, Hph, stat92E, Tre-1, and hop genes, whose RNA interference leads to premature active PGC migration at stages 4–7 of embryogenesis. These genes can be divided into two groups: 1) modulators of JAK/STAT pathway activity inducing PGC migration (stat92E, Tre-1, hop), and 2) myc and Hph involved in epithelial morphogenesis and polarization, i. e. modifying the permeability of the epithelial barrier. Since a depletion of each of these gene products resulted in premature PGC migration, we can conclude that, normally, the somatic environment negatively regulates PGC migration during early Drosophila embryogenesis.

## Abstract (ru)

Клеточная миграция – важный морфогенетический процесс, необходимый на разных этапах ин- дивидуального развития и функционирования организма. Инициация и поддержание состояния движения клеток требуют активации множества факторов, участвующих в регуляции транскрипции, преобразованиях сигналов, адгезивных взаимодействиях, модуляциях мембран и цитоскелета. Однако клеточная миграция за- висит не только от статуса клеток, способных к активному движению, но и от состояния окружающих клеток, с которыми взаимодействуют движущиеся клетки. Окружающие клетки или матрикс не просто формируют субстрат для перемещения, но могут также участвовать в пространственно-временной регуляции миграции. В настоящее время нет точных представлений о генетических механизмах этой регуляции. Чтобы опреде- лить роль клеточного окружения в регуляции индивидуальной клеточной миграции, в настоящей работе мы изучали миграцию клеток зародышевой линии (КЗЛ) в раннем эмбриогенезе Drosophila melanogaster. В нор- ме КЗЛ обособляются на 3-й стадии эмбриогенеза на заднем полюсе эмбриона. Во время гаструляции (6–7-я стадии) КЗЛ в виде консолидированной группы пассивно перемещаются внутрь эмбриона и оказываются в кармане первичной кишки. Далее КЗЛ индивидуализируются, приобретают амебоидную форму, активно перемещаются сквозь эпителий кишки и мигрируют в 5–6-й брюшные сегменты эмбриона, где формируют парные первичные гонады. Мы провели скрининг генов, экспрессирующихся в окружающем КЗЛ эпите- лии в раннем эмбриогенезе и влияющих на их миграцию. Выявили гены myc, Hph, stat92E, Tre-1 и hop, РНК- интерференция которых приводит к преждевременной активной миграции КЗЛ на 4–7-й стадиях эмбриогенеза. Эти гены можно разделить на две группы: модуляторы активности JAK/STAT сигнального каскада, индуцирующего миграцию в КЗЛ, – гены stat92E, Tre-1, hop, и гены, вовлеченные в морфогенез и поляризацию эпителия, т. е. модифицирующие проницаемость эпителиального барьера, – myc, Hph. Так как снижение ко- личества продуктов каждого из этих генов приводило к преждевременной миграции КЗЛ, то можно сделать вывод, что в норме на ранних стадиях эмбриогенеза соматическое окружение негативно регулирует мигра- цию клеток зародышевой линии.

## Introduction

Cell migration is an important morphogenetic process necessary at different stages of development and organism functioning. Large-scale migration of cells occurs during the formation of germ layers, then at the stage of differentiation of organs and tissues (Aman, Piotrowski, 2010; Schumacher, 2019). Also, some differentiated cells retain the ability to migrate when performing their specialized functions (Ratheesh et al., 2015; Barros-Becker et al., 2017; Shapouri-Moghaddam et al., 2018). The initiation and maintenance of cell movement state requires the activation of many factors involved in the regulation of transcription, signal transduction, adhesive interactions, modulation of membranes and the cytoskeleton (Devreotes, Horwitz, 2015). Molecular bases of these processes are evolutionary conserved with high homology in different cell types and different species. Therefore, various aspects of cell migration and the mechanisms of its regulation have been successfully studied in model organisms, both in vivo and in vitro. Drosophila melanogaster embryo represents an excellent model to study these processes (Reig et al., 2014)

Early Drosophila embryo develops as a syncytium. During first 15 min (first stage of embryogenesis), male and female pronuclei fuse and undergo 13 rounds of mitoses. At the third stage of embryogenesis, the first cells are formed. These are primordial germ cells (PGCs) that bud at the posterior pole of the syncytial embryo in the pole plasm region. The rest of the nuclei continues mitoses and acquires cell membranes only at the fifth stage of embryo development during the process of cellularization. During gastrulation, PGCs as a consolidated group are passively internalized from posterior pole to the midgut pocket by the invagination of the embryonic surface. At the tenth stage of embryogenesis, PGCs in the midgut pocket loose cell-cell contacts, individualize and acquire an amoeboid form. At the same time, the process of epithelial to mesenchymal transition (EMT) is activated in midgut primordium cells, which results in partial loss of apical-basal polarity accompanied by diminished intercellular contacts. This allows PGCs to actively move through midgut epithelium and migrate to the region of gonad formation. During active migration, PGCs split up into two groups and coalesce with mesoderm cells in fifth abdominal segment to form paired embryonic gonads (Dansereau, Lasko, 2008; Richardson, Lehmann, 2010).

Earlier, we showed that transcription factor GAGA (GAF), encoded by Trl gene in Drosophila, participates in the regulation of PGC migration during early embryogenesis (Dorogova et al., 2016). Primordial germ cells of Trl mutants, instead of passive translocation as a consolidated group of cells, actively migrate from posterior to the interior of the early embryo. Furthermore, PGCs loose round shape, acquire cytoplasmic protrusions reminiscent of lamellipodia, move chaotically and as a result do not participate in gonad formation. We showed that Trl protein was absent in PGCs, but the effect of their premature migration during early embryogenesis depended on the expression of Trl in somatic cells surrounding PGCs (Dorogova et al., 2016). Current study focuses on the identification of the transcription factor GAF target genes, participating in the regulation of PGC migration by surrounding somatic epithelial cells.

## Materials and methods

D. melanogaster strain Hikone AW – laboratory stock of the Institute of Cytology and Genetics of Siberian Branch of the Russian Academy of Sciences (Novosibirsk, Russia) – was used as a wild type. All other fly strains were obtained from National Institute of Genetics (NIG), Japan, and Bloomington Stocks Center, USA. Stock numbers and corresponding genotypes are represented in Table 1. All strains listed in the Table 1 carry genetic constructs for ectopic RNA interference (RNAi) of corresponding gene. To induce RNAi, flies carrying RNAi construct under the UAS promoter were crossed to flies carrying tub-GAL4, ubiquitously expressing GAL4 transcription factor that specifically binds to UAS sequence and induces expression. Flies were maintained on a standard agar/corn media at 25 °С.

**Table 1.**
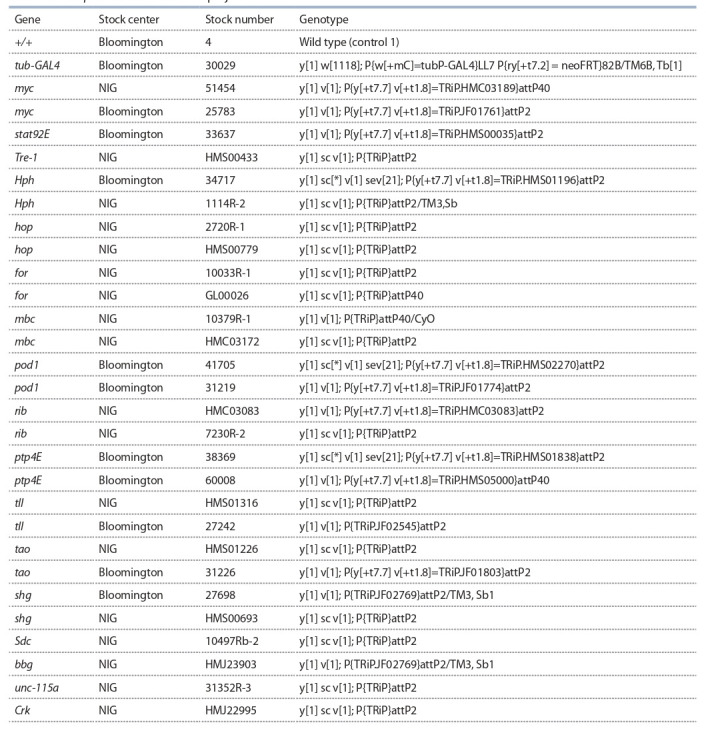
Drosophila stocks used in the project

Dissection, fixation and immunostaining of embryos were described earlier (Dorogova et al., 2014). Mated females were let to lay eggs overnight, after which embryos were collected, fixed and stored in methanol at –20 °С. For the analysis, embryos were rehydrated and immunostained. Following antibodies were used: primary rabbit anti-Vasa (1:30, SC-30210, Santa Cruz Biotechnology), secondary anti-rabbit Alexa 568 (1:300, Molecular probe, A-11011). After incubation with antibodies embryos were stained with DAPI (2 mg/ml in 1 × PBS, pH 7.4) for 5 min and mounted in Mowiol containing 10 % DABCO. For each combination (RNAi of gene induced by ubiquitous tub-GAL4) 400–500 embryos of different stages were analyzed. Wild type flies Hikone AW, tub-GAL4 flies and fly strains listed in Table 1 in the absence of RNAi induction were used as controls. 100–200 embryos at different stages were analyzed for each control group. Slides were analyzed using AxioImager Z1 equipped with ApoTome (Zeiss, Germany) and AxioCam MR (Zeiss).

Bioinformatic analysis was performed using following databases: FlyExpress, Fly-FISH, Berkeley Drosophila Genome Project and DRoID. By comparing results from the above databases, we obtained a list of genes expressed at the desired stage of embryogenesis and potentially regulated by GAGA transcription factor. The analysis of non-canonical binding sites was performed using SITECON (Omelina et al., 2011). Regulatory elements 500 base pairs upstream of the transcription start site were used for the analysis.

## Results

In our previous work we showed that mutants for Trl gene, which encodes transcription factor GAGA (GAF), had defects in PGC migration during embryogenesis (Dorogova et al., 2016). The absence of GAF resulted in premature transepithelial movement of PGCs from the posterior pole to the interior part of the embryo. We found that the effect of early PGC migration depended on zygotic expression of Trl in somatic cells of posterior embryo pole. Since GAF is a transcription regulator, we reasoned that its effect on PGC migration is mediated via its target genes. To date, experimental data exist on binding of GAF with promoters of about 300 genes (van Steensel et al., 2003; Omelina et al., 2011) participating in a wide range of fundamental cell processes.

In this work, to determine the role of cell environment in regulating the individual cell migration during early embryo development, we conducted a screen for genes that expressed in somatic cells, surrounding PGCs during 4–6 stages of embryogenesis. This pattern is characteristic of Trl expression at the indicated stages, based on published data and Berkley Drosophila Genome Project database (https://insitu.fruitfly.org/cgi-bin/ex/insitu.pl). The set of 81 genes was identified that included stat92E, hop, Trl and Tre-1 – well known regulators of cell migration (Kunwar et al., 2003, 2008; Li et al., 2003; Sheng et al., 2009; Dorogova et al., 2016). According to database DroID, 67 % of the selected genes represent potential targets of transcription factor GAF. Therefore, we analyzed these genes for the presence of GAF binding sites using SITECON (Omelina et al., 2011). Regulatory elements 500 base pairs upstream of the transcription start site were used for the analysis. As a result, we found 39 potential GAF binding sites of the GAGnGAG type, and 68 of the GAGnnnGAG type in the region –500…+1. As an initial verification of identified binding sites functionality we analyzed the presence of GAF binding peaks using ModEncode database (Embryo_0_12h_GAF_ChIP_chip; http://www.modencode.org/). For the majority of genes (32 from 39 for GAGnGAG sites and 62 from 68 for GAGnnnGAG sited) GAF binding was observed during embryogenesis (for two genes, the data were absent).

**Fig. 1.**
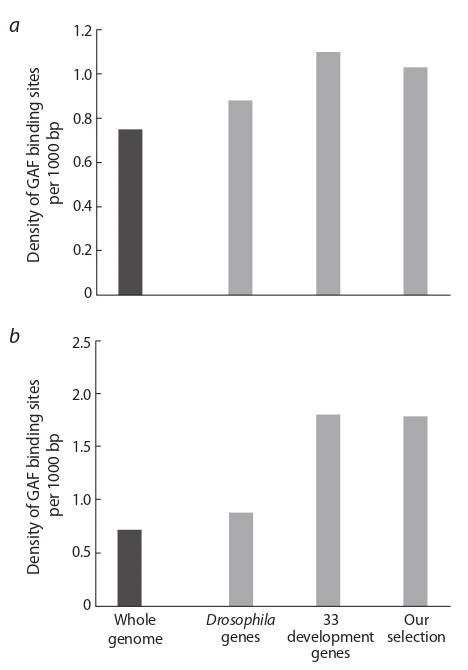
The distribution density of potential GAF binding sites (per 1000 nucleotides) of the GAGnGAG (a) and GAGnnnGAG (b) types in various gene sets. The black column is the density of GAF binding sites throughout whole Drosophila genome; the light gray columns are the density of GAF sites in the promoter regions (–500…+1) of various gene sets: 6947 random Drosophila genes, 33 Drosophila development genes (Omelina et al., 2011), and our set of genes which expressed in the epithelial cells at the posterior pole of 4–6 stage embryos.

Interestingly, the density of GAF binding sites distribution in –500…+1 region of genes expressing in early embryogenesis in somatic cells surrounding PGCs, was not random (Fig. 1) and exceeded the average density of such sites throughout whole Drosophila genome. For example, density of GAGnnnGAG sites for our gene set was more than twice higher compared to the genome-wide distribution density (see Fig. 1, b). What is more intriguing is that the density of GAF binding sites in our set of genes was similar to that of Drosophila development genes. Our gene set consisted of genes expressed in early embryogenesis, however, most of them were not early development genes.

**Fig. 2.**
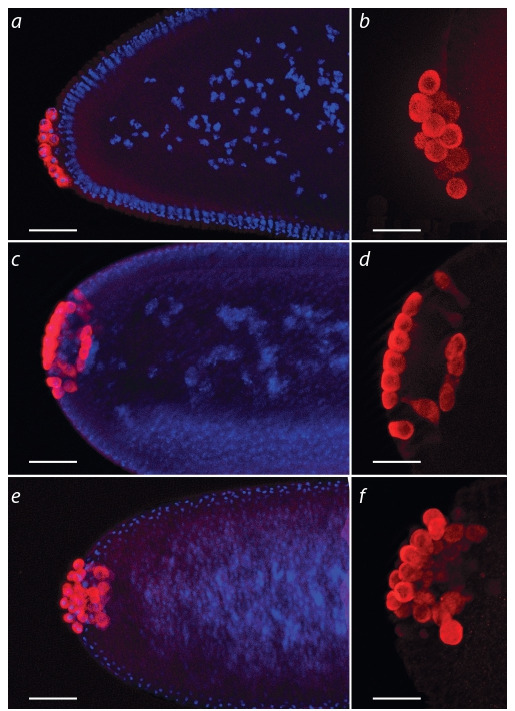
Primordial germ cells at 4–5 stage of embryonic development in control (a, b), in tub-GAL4/UAS-myc-RNAi (c, d ) and tub-GAL4/UAS-Hph- RNAi (e, f ). a, b – primordial cells are located at the posterior pole of the embryo and have a spherical shape typical for this stage; c, d – premature germ cells migration in tub-GAL4/UAS-myc-RNAi embryos; e, f – premature germ cells migration in tub-GAL4/UAS-Hph–RNAi embryos. The nuclei are stained with DAPI, Germ cells – antibodies to the VASA protein. Scale: a, c, e – 30 μm; b, d, f – 50 μm.

Next we analyzed the migration of PGCs during RNAi of genes from our gene set. To induce RNAi we used the GAL4/UAS system with ubiquitous tub-GAL4 driver (Table 2, Fig. 2). For many genes, transgenic fly stocks with RNAi constructs against different parts of the gene were available. In such cases we used all available stocks in independent experiments. Hikone AW wild type stock, tub-GAL4 stock and stocks carrying UAS-RNAi (in the absence of GAL4 induction) were used as controls. In all controls premature PGC migration was not observed.

**Table 2.**
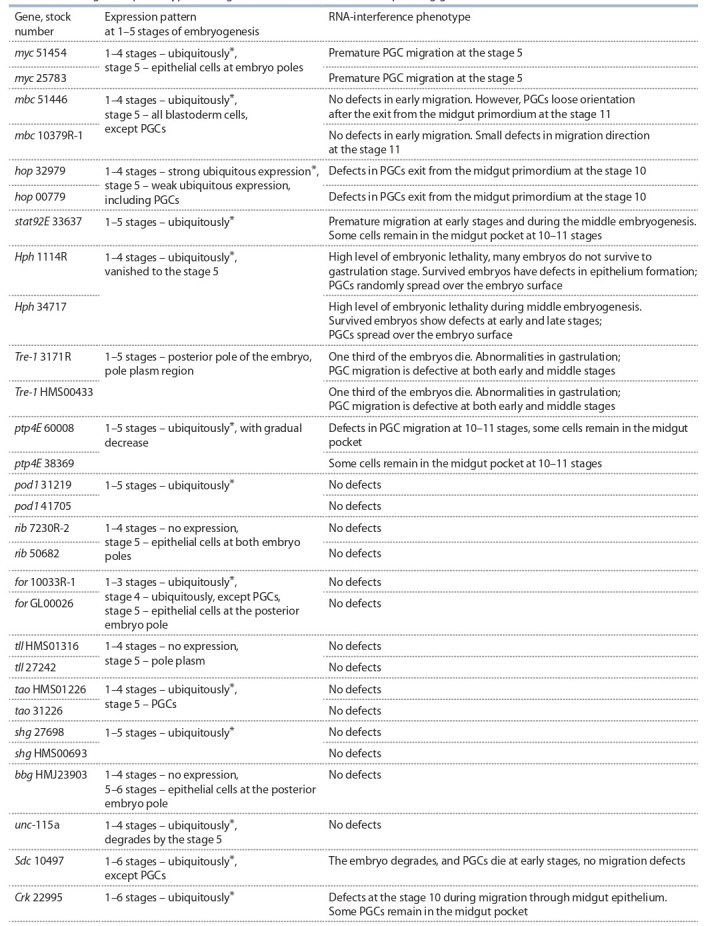
PGC migration phenotypes during RNA interference of the corresponding gene * Ubiquitously – means the localization of mRNA of the corresponding gene throughout the whole volume of the embryo including pole plasm region at 1–3 stages and the formed PGCs at 4–6 stages (unless otherwise indicated).

Premature PGC migration in early embryogenesis was observed during RNAi of genes myc (~90 %, n* = 60; * hereinafter n – the number of viewed embryos of genotype tub-GAL4/UAS–RNAi at 4–6 stage of embryogenesis), Hph (~90 %, n = 45), stat92E (~90 %, n = 50), Tre-1 (~70 %, n = 40) and hop (~40 %, n = 40). In addition to defects in migration at early stages, RNAi of stat92E, Tre-1 and hop genes also resulted in PGC migration defects during middle embryogenesis, at stages 10–11. Several genes, shg, hop, mbc, Hph, ptp4E and Crk, were found to be involved in PGC migration through primordium midgut epithelium at stages 10–11. We did not reveal any effect of RNAi of tll, tao, for, bbg, unc-115a, Sdc, pod1 and rib genes on PGC migration in embryogenesis.

## Discussion

We discovered the premature PGC migration phenotype that depended on the transcription factor GAF (encoded by Trl gene in Drosophila) expression in somatic cells surrounding PGCs (Dorogova et al., 2016). This phenomenon prompted us to search for other genes regulating PGC migration in early embryogenesis. Interestingly, 67 % of genes that expressed in epithelial cells surrounding PGCs at 4–6 stages of embryo development are potential targets of transcription factor GAF, which suggests its active role in PGC migration regulation. We compared our data with data on transcriptional regulation of other types of migrating cells in Drosophila embryo (Bae et al., 2017): caudal visceral mesoderm (precursors of longitudinal muscles of the gut, they migrate collectively), and hemocytes (the Drosophila equivalent of blood cells, they migrate individually). Among 73 genes common for somatic migrating cells, the expression of 64 genes (88 %) can be regulated by GAF. Also, it is worth mentioning that the distribution density of GAF binding sites in the promoters of genes from our gene set was two times higher than the random density, and corresponded to the density in early development genes (Omelina et al., 2011). Therefore, we propose that GAF regulates not only embryonic cell migration, but also early Drosophila development in general.

Since genes participating in the control of embryonic cell migration were enriched with targets of GAF transcription factor, we further selected genes involved in PGC migration regulation from surrounding somatic cells. We analyzed 17 genes from obtained gene set for their effect on PGC migration, and revealed a number of genes that negatively regulated this process. We showed that RNA interference of myc, Hph, stat92E, Tre-1 and hop genes resulted in premature transepithelial migration of PGCs in early embryogenesis. The involvement of last three genes was known before: hop (hopscotch) and stat92E encode JAK kinase and transcription factor STAT92E (Signal Transducer and Activator of Transcription), respectively. The products of both of these genes activate evolutionary conserved JAK/STAT signaling pathway that induces cell migration in Drosophila, mice and humans (Li et al., 2003; Silver et al., 2005). Premature PGC migration that we observed during RNAi of hop is in agreement with J. Li and colleagues (2003) who showed that hop expressed only in somatic cells of the embryo, and its mutations resulted in hyperactivation of STAT92E and premature PGC migration. It should be noted that to initiate migration, JAK/ STAT pathway needs to be activated in PGCs themselves. During early embryogenesis, JAK/STAT signaling cascade in PGCs is activated by Tor kinase (Li et al., 2003). In somatic cells surrounding PGCs, this cascade is activated by JAK kinase and seems to control the reorganization of cytoskeleton and polarization of epithelial cells.

The role of JAK/STAT in epithelium morphogenesis and polarization has been shown for many tissues including Drosophila embryonic intestine (Josten et al., 2004). Tre-1 is also a part of JAK/STAT pathway and it encodes G protein-coupled receptor (GPCR) – transmembrane chemoattractant receptor (Kunwar et al., 2003, 2008; Sheng et al., 2009). The activation of Tre-1 initiates significant intracellular rearrangements – the cell changes its polarization and cytoskeleton regulation, which altogether creates the conditions for active cell movement. It has been shown that Tre-1 is also important for cell polarization at early stages of embryo development (Richardson, Lehmann, 2010). We hypothesize that the RNA interference of Tre-1 leads to the weaker polarization of epithelial cells and, as a result, to increased permeability of epithelium for migrating cells.

The most interesting for us is the result on the role of myc and Hph in the regulation of PGC migration. myc (or dm – diminutive) encodes the well-known transcription factor homologous to vertebrate Myc protooncogene, which is important for cell proliferation and growth. Surprisingly, recent genetic screen for modulators of tumor invasion identified Myc as a negative regulator that blocked tumor invasion and metastasis (Ma et al., 2017). Ectopic expression of human cMyc potently suppressed JNK-dependent cell invasion and migration in both Drosophila and lung adenocarcinoma cell lines (Ma et al., 2017). The authors showed that Myc, together with its transcriptional partner, upregulated the expression of tyrosine kinase рuc (рuckered), which, on the one hand, is involved in polarization and morphogenesis of epithelial cells, and, on the other hand, inhibits JNK signalling pathway critical for tumor invasion and cell migration (Ma et al., 2017). Therefore, the decrease of Myc in somatic cells might promote PGC migration due to the increase in the permeability of surrounding epithelium.

Hph (HIF prolyl hydroxylase) encodes HIF prolyl- 4-hydroxylase which acts as an oxygen sensor. Mutations in Hph result in defects in embryonic tracheal development. In Drosophila oogenesis, Hph regulates border cell migration speed in a dose-dependent manner: overexpression of Hph increases border cell migration, whereas Hph depletion has opposite effect (Doronkin et al., 2010). In addition, Hphmutant mosaic clones show diminished expression levels of slbo (slow border cells) – key regulator of border cell migration and shg (shotgun) – gene encoding cell adhesion protein DE-cadherin (Doronkin et al., 2010)

To sum up, we revealed negative regulators of PGC migration in early embryogenesis that falls into two categories: modifiers of JAK/STAT signaling pathway activity that induces cell migration in many organisms, and genes involved in epithelium polarization and morphogenesis. Genes in the first group normally reduce the invasiveness of migrating primordial germ cells, whereas genes in the second group are responsible for the impermeability of the epithelial layer.

## Conclusion

In this work we performed screen for genes involved in primordial germ cell migration during early embryo development. A key feature of our screen was that we searched for the regulators not in migrating PGCs themselves but in surrounding somatic cells. We identified five genes, myc, Hph, stat92E, Tre-1 and hop, whose RNA interference resulted in a premature PGC migration in early embryogenesis. The premature migration phenotype demonstrates that surrounding cells, somatic cells in particular, not only form a substrate for PGCs movement, but can also actively regulate the migration itself. This aspect of migration regulation is poorly studied and far from being well-understood. Nevertheless, it requires further detailed studies because of homology with other types of individual cell migration, including cell migration during metastasis.

## Conflict of interest

The authors declare no conflict of interest.
